# Study of half metallicity, structural and mechanical properties in inverse Heusler alloy Mn_2_ZnSi_(1−*x*)_Ge_*x*_ and a superlattice

**DOI:** 10.1039/c9ra06903h

**Published:** 2019-11-11

**Authors:** M. Ram, A. Saxena, Abeer E. Aly, A. Shankar

**Affiliations:** Department of Physics, North-Eastern Hill University Shillong India-793022 atulnehu@yahoo.co.in; Condensed Matter Theory Research Lab, Department of Physics, Kurseong College Darjeeling Kurseong India-734203 amitshan2009@gmail.com; Basic Science Department, El Salam Institute for Engineering and Technology Cairo Egypt abeerresmat782000@yahoo.com

## Abstract

The electronic and magnetic properties of Mn_2_ZnSi_(1−*x*)_Ge_*x*_ (*x* = 0.0, 0.125, 0.25, 0.375, 0.5, 0.625, 0.75, 0.875, and 1.0) inverse Heusler alloys and Mn_2_ZnSi/Mn_2_ZnGe superlattice have been investigated using first-principles calculations. All these alloys are stable in the fcc magnetic phase and satisfies the mechanical and thermal stability conditions as determined from the elastic constants and negative formation energy. The spin-polarized electronic band structures and the density of states indicate half-metallicity with 100% spin polarization at the Fermi energy level for *x* = 0.0, 0.125, 0.25, 0.50, and 1.0, with the integral values of the total magnetic moments per formula unit at their equilibrium lattice constants, following the Slater–Pauling rule. The electronic properties and the magnetic moments are mostly contributed by two Mn atoms and are coupled anti-parallel to each other, making them ferrimagnetic in nature. The presence of the half-metallic bandgap with an antiparallel alignment of Mn atoms makes these Heusler alloys a potential candidate for spintronic applications.

## Abstract 2

The electronic and magnetic properties of Mn_2_ZnSi_(1−*x*)_Ge_*x*_ (*x* = 0.0, 0.125, 0.25, 0.375, 0.5, 0.625, 0.75, 0.875, and 1.0) inverse Heusler alloys and Mn_2_ZnSi/Mn_2_ZnGe superlattice have been investigated using first-principles calculations.

## Graphical abstract

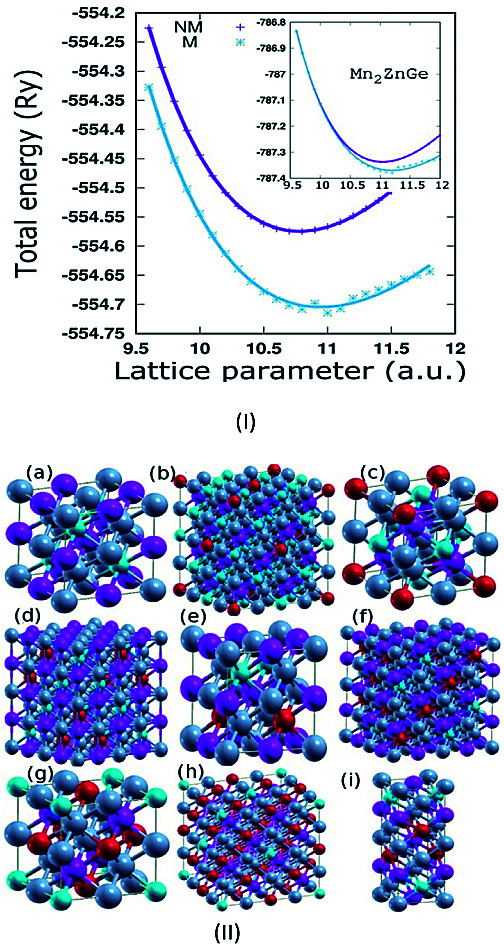

## 1. Introduction

Half-metals (HMs) are a type of materials where we observe a metallic nature for one kind of electron spin and a semiconducting gap at the Fermi energy level (*E*_F_) for the other electron spin, thus having 100% spin-polarized electrons at *E*_F_. Such materials are a good source of spin-polarized electrons for applications in the trending field of spintronics, which manipulates the spin degrees of freedom of electrons in addition to their charges, and are finding large applications in new phenomena such as giant magnetoresistance,^1,2^ tunnelling magnetoresistance, and magnetic tunnel junctions.^3^ Although the half-metallic properties have been observed in systems like oxides, manganites, double perovskites, and pyrites,^4–8^ it is the half-metallic Heusler alloys that are getting more preference over others due to the similarity in the crystal structure with the primarily used binary semiconductors (GaAs) and comparatively high Curie temperature (*T*_c_) (Co_2_FeSi, *T*_c_ = 1100 K).^9,10^ The half-metal (HM) nature has been predicted by several authors in a variety of Heusler alloys,^11–14^ including full Heusler alloys with a general formula of X_2_YZ (where X and Y are transition metals and Z is an sp block element), from their theoretical and experimental studies, with Co_2_MnSi(Ge) being the first member.^15,16^

The Mn-based Heusler alloys in cubic and tetragonal phases have gained much interest among the Heusler alloys in the field of shape memory,^17^ giant topological Hall effect,^18^ spin-transfer torque,^19^ and large exchange bias^20,21^ owing to their stable half-metallicity with 100% polarization at *E*_F_ and high *T*_c_ with ferri/ferro-magnetism.^22–26^ A compensating ferrimagnetic order of Mn also results in low saturation magnetization in these alloys,^27^ which results in the reduction of power loss due to the absence of stray fields; also, the absence of inversion symmetry evolves interesting phenomena such as non-collinear magnetism, topological Hall effect, and skyrmions that are absent in the centrosymmetric Heusler structures like Mn_2_YGa (Y = Ti, V, Cr).^27^ A significant amount of theoretical and experimental investigations are in progress to explore the physical properties of Mn-based candidates^28^ as observed from literature, but new Mn-based Heusler alloys are waiting to be discovered with better and untapped properties. Although detailed studies on Mn_2_ZnSi(Ge) have been reported by previous authors,^29–32^ there exist some discrepancies in their reports. In this context, Wei *et al.*^31^ have estimated a smaller lattice constant of 5.75 Å for Mn_2_ZnGe as compared to that of Mn_2_ZnSi (5.80 Å),^29,30^ which could be due to the larger atomic size of Ge than that of Si. A similar anomaly was also communicated in their electronic properties.^29,31^ Lie *et al.*^32^ suggested the occurrence of an energy band gap of 0.52 eV at the majority spin, inconsistent with the energy gap of 0.48 eV at the minority spin channel, as reported by Bhat *et al.*^30^ Similarly, the asymmetric electronic structure has been reported for isoelectronic Mn_2_ZnSi and Mn_2_ZnGe^31^ in contrast to the symmetric profile of analogous systems like Mn_2_FeZ (Z = Al, Ga, Si, Ge, and Sb),^33^ Mn_2_VZ (Z = Al, Ge),^34^ and Mn_2_CoZ (Z = Al, Ga, Si, and Ge).^35^

To remove the above anomaly observed in the physical properties of Mn_2_ZnSi(Ge), we performed a detailed investigation of the structural and electronic properties using the well-known density functional theory (DFT). Since there are no experimental reports available for the title compounds, the results obtained for Mn_2_ZnSi(Ge) were validated by systematically doping the Mn_2_ZnSi system with Ge and subsequently comparing with the available theoretical data. A similar study of the substitution of Si by Ge in Fe_2_MnSi_(1−*x*)_Ge_*x*_ was studied by Hamad *et al.*,^36^ where the valence bands and the conduction bands shift to higher energies for 0.75 concentration of Ge, causing the bands to cross *E*_F_ and resulting in the loss of half-metallicity. A similar behaviour for 0.75 concentration of Ge could also be expected and it motivated us to do similar studies on more finer concentrations of Ge, *i.e.*, Mn_2_ZnSi_(1−*x*)_Ge_*x*_ (*x* = 0.0, 0.125, 0.25, 0.375, 0.5, 0.625, 0.75, 0.875, and 1.0) to understand and verify whether such similar properties, due to the symmetry in the concentration of Si and Ge, exist.

It is also intended to construct a superlattice of Mn_2_ZnSi/Mn_2_ZnGe along the [001] direction of the parent (Mn_2_ZnSi) fcc lattice. The half-metallicity and the Slater–Pauling rule in the superlattice of two Heusler alloys remained unaffected by the crystal directions, as shown by Azadani *et al.* along [001], [110], and [111] direction with various thickness.^37^ Moreover, they have reported the presence of induced uniaxial magnetocrystalline anisotropy in the superlattice, which is prohibited in L2_1_ and C1_b_ structure of Heusler alloys due to their symmetry. These superlattices are also reported to be efficient in reducing the thermal conductivity (*κ*) in the thermoelectric materials by reducing the phonon contribution to *κ*, which is achieved by the additional photon scattering at the interface of the superlattice.^38^

## 2. Computational details

The DFT based plane-wave pseudopotential (PW-PP)^39^ and full potential-linearized augmented plane-wave (FP-LAPW) methods^40^ were employed to investigate the results presented in this manuscript. The basis set was expanded in terms of plane waves in PW-PP, whereas in the FP-LAPW method, space was divided into non-overlapping muffin-tin (MT) spheres centred on the atomic sites and in an interstitial region (IR). The basis set inside the MT sphere consists of a linear combination of radial functions times spherical harmonics, whereas it consists of plane waves in the IR. The Kohn–Sham orbitals were described by expanding the kinetic energy with the cut-off value of 50 Ry in PW-PP and charge-density cut-off value of 500 Ry. The electron–ion interactions were described by the Vanderbilt ultrasoft potentials. The fermionic occupations were described by the Marzari–Vanderbilt^41^ scheme of smearing, with a value of 7.4 × 10^−3^ Ry. The energy convergence was achieved in the FP-LAPW method by expanding the plane wave functions in IR, with a cut off *R*_MT_ × *K*_max_ = 8, where *R*_MT_ denotes the smallest muffin-tin sphere radius and *K*_max_ gives the maximum value of the wave vector (*K*) in wave expansion. Different MT sphere radii (*R*_MT_) were used with the value of 2.44 a.u., 2.40 a.u., 2.30 a.u., and 2.40 a.u. for Mn, Zn, Si, and Ge, respectively. For the non-spherical contributions, charge density and the potential inside the MT spheres were expanded up to *l*_max_ = 14, while the charge density and the potential were expanded as a Fourier series with the wave vectors up to *G*_max_ = 14. Integration over the Brillouin zone was carried out on a grid of 16 × 16 × 16 with automatically generated k-points following the convention of Monkhorst and Pack^42^ centred at *Γ*-point. The effect of exchange-correlation functional was treated with the Perdew–Burke–Ernzerhof scheme of generalised gradient approximation (PBE-GGA).^43^

## 3. Results and discussions

### 3.1 Crystal structure and structural properties

**Fig. 1:**
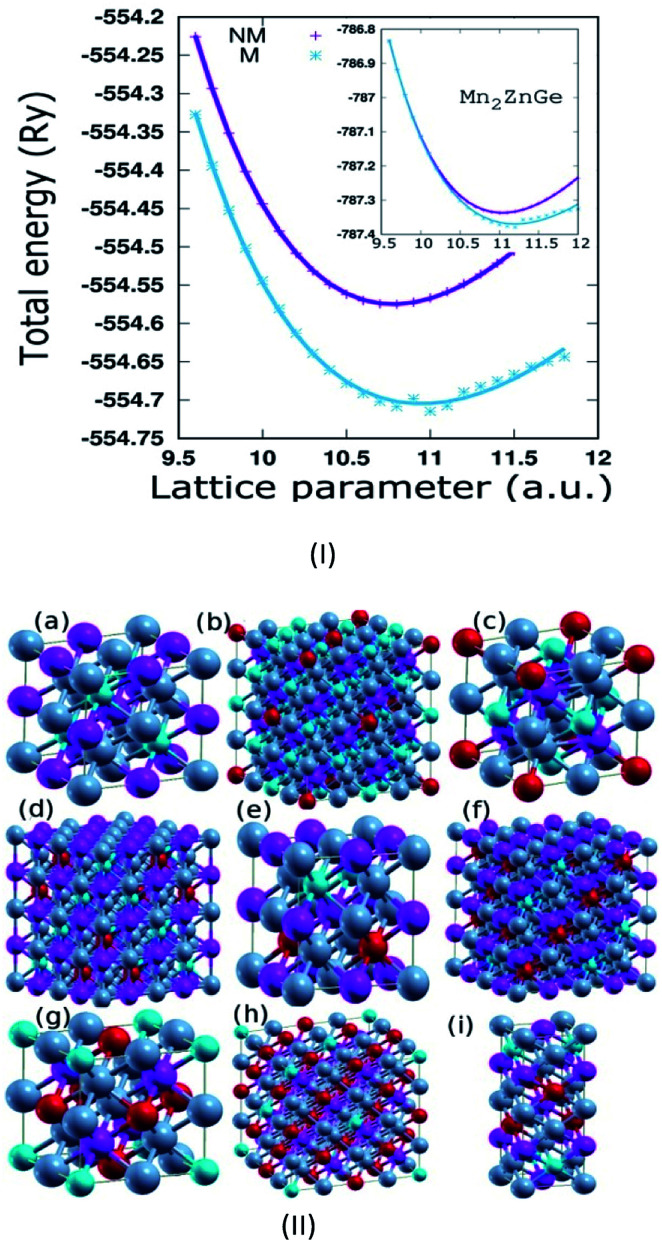
(I) The total energy as a function of lattice constant of Mn_2_ZnSi (inset for Mn_2_ZnGe) for non-magnetic and magnetic phases along with the fitted Murnaghan's equation of state (Solid lines). (II) Crystal structure of (a) Mn_2_ZnSi (Ge) & Ge doped with (b) 0.125, (c) 0.25, (d) 0.375, (e) 0.5, (f) 0.625, (g) 0.75, (h) 0.875 and (i) the superlattice of Mn_2_ZnSi/Mn_2_ZnGe respectively (colour scheme: Mn = grey, Zn = purple, Si = blue and Ge = red).

Mn_2_ZnSi(Ge) crystallizes in the fcc structure of prototype Hg_2_CuTi-type (space group *F*4̄3*m*),^11^ as shown in Fig. 1, where Mn occupies the position (0, 0, 0) and (1/4, 1/4, 1/4) labelled as MnI and MnII, respectively, while Zn occupies (1/2, 1/2, 1/2) and Si(Ge) occupies (3/4, 3/4, 3/4) of a unit cell, with the atomic sequence being Mn–Mn–Zn–Si(Ge).^31^ The non-magnetic (NM) and magnetic (M) phases of the sample materials were optimized to verify the most stable configuration. The lattice constant (*a*) *versus* the total energy fitted into empirical Murnaghan's equation of state is shown in Fig. 1(I). We can conclude that the sample materials crystallize in the magnetic phase ground state. The optimized *a* for Mn_2_ZnSi (5.79 Å) is in qualitative agreement with the previous report (5.80 Å); however, there exist discrepancy in the results for Mn_2_ZnGe. We obtained an optimized *a* = 5.93 Å for Mn_2_ZnGe, but Wei *et al.*^31^ have mentioned that Mn_2_ZnGe reflects HM behaviour, with the value of *a* ranging from 5.69 to 5.80 Å and an equilibrium lattice constant of 5.75 Å. It can also be mentioned here that slightly higher *a* is expected for Mn_2_ZnGe as compared to Mn_2_ZnSi, due to the addition of Ge having a bigger atom radius, as observed in analogous Fe_2_MnZ (Z = Si, Ge, and Sn) reported by Jain *et al.*,^44^ where the crystal size increases with the replacement of Z from Si to Sn.

For doping with the Ge concentration of *x* = 0.25, 0.5, and 0.75, a 1 × 1 × 1 unit cell of Mn_2_ZnSi, with 16 basis was used, giving 4 atomic positions to Si. All these 4 positions were equivalent due to symmetry, and any Si atom(s) can be replaced by Ge to give 0.25, 0.5, and 0.75 concentrations (Fig. 1(II)). However, the doping by 0.125, 0.375, 0.625, and 0.875 concentration of Ge needs a 2 × 2 × 2 supercell with 32 atom basis, giving 8 atomic positions to Si. Now, for the Ge concentration of 0.125, any 1 Si atoms can be replaced, since all the positions are equivalent due to symmetry. However, for Ge concentration of 0.375, 3 Si atoms have to be replaced, which can be achieved in 56 ways. However, symmetry reduces the number of structures, giving a total of 5 types of different structures. For the Ge concentrations of 0.625 and 0.875, the different types of structures were obtained by exchanging the atomic positions between Si and Ge for 0.375 and 0.125, respectively. Subsequently, we computed the ground state energies of all the possible structures for each doping, and the structure with minimum energy was considered for further studies. The ground state optimized *a* of the 1 × 1 × 1 unit cell for all doping concentrations along with the calculated *a*_v_ from the Vegards law (eqn. (1)) are given in Table 1. The optimized values were found to be in qualitative agreement with that obtained from the Vegards law, which is given as:

1 *a*_*x*_ = 5.793(1 − *x*) + 5.926(*x*)

where *x* is the doping concentration of Ge with *a*_*x*_ as the corresponding Vegards lattice constant and 5.793 and 5.926 being the optimized lattice constants (in Å) of Mn_2_ZnSi and Mn_2_ZnGe, respectively.

**Table 1:** The calculated equilibrium cell dimensions (*a*), Vegards lattice constant (*a*_v_), difference between *a* and *a*_v_, and formation energy (*E*_form_) (Note: for the superlattice, it is *a*_s_ = *a*/√2 value, *a* of fcc lattice, and *c*_*s*_ of superlattice)

Ge concentration (*x*)	Optimized *a* (Å)	Vegards *a*_v_ (Å)	Difference between *a* and *a*_v_	*E*_form_ (eV)
0.0	5.793	—	—	−2.866
0.125	5.798	5.809	−0.001	−2.830
0.25	5.814	5.826	−0.012	−2.738
0.375	5.837	5.843	−0.006	−2.639
0.5	5.861	5.86	0.001	−2.625
0.625	5.883	5.876	0.007	−2.532
0.75	5.895	5.893	0.002	−2.471
0.875	5.913	5.909	0.004	−2.417
1.0	5.926	—	—	−2.374
Superlattice	4.091, 11.57	—	—	−2.106

For the construction of the superlattice of [Mn_2_ZnSi]_*n*_/[Mn_2_ZnGe]_*n*_ (for notation, refer 45), we used the technique used by Culbert *et al.*^46^ and Tirpanci *et al.*^45^ As described earlier in section 3.1, the structure of X_2_YZ inverse Heusler alloys with space group *F*4̄3*m* is generally described as fcc with four atom basis. However, it can also be considered as a bcc layered structure along the [001] direction with two atoms in a layer (Fig. 1 of ref. 46). The lattice constant (*a*_s_/*b*_s_, subscript s for superlattice) of the layered superlattice and the lattice constant (*a*) of the parent fcc are related as *a*_s_ = *b*_s_ = *a*/√2, while *c*_s_ depends on the *n* value. In the present case with *n* = 1, the sequence of the atomic layer in the periodic superlattice is MnZn–MnSi–ZnMn–GeMn–MnZn–MnGe–ZnMn–SiMn. For example, for the superlattices of lattices A and B, Tirapanci *et al.*^45^ took the lattice constant of the superlattice as the average of the lattice constants of A and B, whereas in the present study, we performed the volume optimization of the superlattice, which gives us *a*_s_ = *b*_s_ = *a*/√2 = 4.091 Å, and *a* = 5.786 Å for the parent fcc lattice. The *c* value (Table 1) is exactly double of *a*, and this exactly corresponds to a bcc structure for *n* = 1. However, an intermediate *a* value between that of Mn_2_ZnSi and Mn_2_ZnGe was expected.

In the present study, all the structures obtained were optimized by using the force minimization method, and their stability was verified by calculating their formation energy (*E*_form_) per atom. The formula *E*_form_ per atom of full Heusler alloys as given in ref. 47 and 48 was modified in our case Mn_2_ZnSi_(1−*x*)_Ge_*x*_ as2
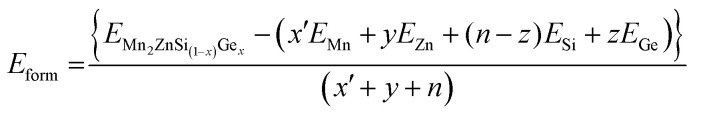
where *E*_Mn_2_ZnSi_(1−*x*)_Ge_*x*__ is the ground state equilibrium energy of Mn_2_ZnSi_(1−*x*)_Ge_*x*_ compounds and *E*_Mn_, *E*_Zn_, *E*_Si_, and *E*_Ge_ represent the equilibrium energy of individual Mn, Zn, Si, and Ge atoms, respectively, in the solid-state. *x*′, *y*, (*n*−*z*), and *z* are the number of Mn, Zn, Si, and Ge atoms in the unit (or super) cell, respectively. Here, *n* is the total number of Z atoms in the unit (or super) cell. The obtained negative formation energy, presented in Table 1, indicates the thermal stability of the whole series of compounds and the possibility of their synthesis.

### 3.2 Mechanical properties

It is also very important to determine the elastic constants of a material to understand their significant properties in the solid-state, such as interatomic potential, mechanical deformation and stress, specific heat, and Debye temperature. The nature of the structural transitions can also be understood by observing the behaviour of parent phases near transitions. Hence, we calculated independent elastic constants (*C*_*ij*_) using the volume conservation technique,^49^ in which the strain was chosen in such a way that the total volume of the system remains constant. The cubic symmetry of the crystal reduces the total number of independent elastic constants from 21 to 3, namely, *C*_11_, *C*_12_, and *C*_44_. The mechanical stability of the structure, after the deformation of a cubic crystal with the *F*4̄3*m* space group, were verified by the expression *C*_11_ > 0, *C*_11_ − *C*_12_ > 0, and *C*_11_ + 2*C*_12_ > 0.^44^ The calculated values of *C*_*ij*_ are given in Table 2, and the values are found to fulfil the above stability conditions.

**Table 2:** The independent constants (*C*_*ij*_), Bulk modulus (*B*), Shear Modulus (*G*), Young's modulus (*Y*) in GPa, Debye temperature (*Θ*_D_) in *K*, Poisson's ratio (*v*), and Anisotropy factor (*A*)

Ge doping (*x*)	*C*_11_	*C*_12_	*C*_44_	*B*	*G*	*Y*	*Θ*_D_	*ν*	*A*
0.0	252.06	102.45	168.89	152.32	121.81	288.53	594.75	0.18	2.26
0.125	205.35	153.3	76.75	170.65	49.79	136.14	385.64	0.37	2.95
0.25	148.79	101.09	112.82	116.99	61.25	156.44	415.99	0.28	4.73
0.375	212.38	185.22	97.14	194.68	45.57	126.82	359.41	0.39	7.32
0.50	202.24	182.44	104.28	189.11	44.04	122.59	349.76	0.38	10.59
0.625	132.97	102.97	105.69	112.97	50.16	131.09	364.55	0.31	7.05
0.75	95.58	48.06	67.92	63.89	44.61	108.56	339.30	0.22	2.86
0.875	185.19	148.6	108.46	160.79	54.45	146.78	379.38	0.35	5.93
1.0	241.35	119.81	195.33	160.33	122.54	292.98	548.33	0.19	3.21

We also computed other parameters, as described in Table 2, from independent elastic constants. The isotropic *B* and *G* values for the samples were estimated from the Voigt–Reuss–Hill (VRH) approximation,^50–54^ where the *B* value defines the hardness of a material, and for pure compounds (*x* = 0.0 and 1.0), it is comparable to that of Mn_2_ZrSi (*B* = 187.015 GPa)^55^ and Mn_2_ZrGe (*B* = 175.478 GPa)^55^ and was found to be highest for 0.375 concentration of Ge among the studied alloys. Similarly, the analysis of Table 2 for resistant to plastic deformation, *i.e.*, the value of *G* also suggest that Mn_2_ZnSi has more resistance as compared to analogous Mn_2_ZrSi (*G* = 80.249 GPa),^55^ Mn_2_ZrGe (*G* = 71.088 GPa),^55^ Fe_2_MnSi (*G* = 73 GPa),^44^ and Fe_2_MnGe (*G* = 86 GPa),^44^ as the resistance becomes lower with the further addition of Ge and again becomes higher for *x* = 1.0. The Young's modulus (*Y*) is a measure of the stiffness of a material, and the higher the Young's modulus value, the stiffer is the material. Typically, pure elements are stiffer than the doped ones, and on comparing with analogous Mn_2_ZrSi (*Y* = 210.622 GPa),^55^ Mn_2_ZrGe (*Y* = 187.189 GPa),^55^ Fe_2_MnSi(*Y* = 198 GPa),^44^ and Fe_2_MnGe (*Y* = 202 GPa),^44^ it was observed that both of our sample materials are the stiffest of all.

It is known that Pugh's ratio (*B*/*G*) determines the brittleness or ductility^56,57^ of a material with its critical value of 1.75, and the nature of atomic bonding present in the material can be verified from its critical value of 0.26 for the Poisson's ratio (*ν*).^58^ We can note from Table 2 that both Mn_2_ZnSi and Mn_2_ZnGe are brittle in nature with the directional covalent type of bonding, but the partial doping of Ge somehow make these alloys ductile in nature. The probability of developing microcracks or defects during the growth process of the crystal was expected for these materials, as observed from their Zener anisotropy factor (*A*),^59^ which is much greater than unity. The thermodynamic property can also be studied from the elastic parameters at Debye temperature (*Θ*_D_) and low temperature, with the crystal vibration being the acoustic type, where *Θ*_D_, estimated from the elastic constant, was expected to describe their real value. It is evident from their values that *Θ*_D_ decreases with the replacement of Si by Ge with a bigger atomic radius, and a similar feature was also observed for isotropic Fe_2_MnZ (Z = Si, Ge, and Sn).^44^

### 3.3 Electronic properties and magnetic moments

**Fig. 2:**
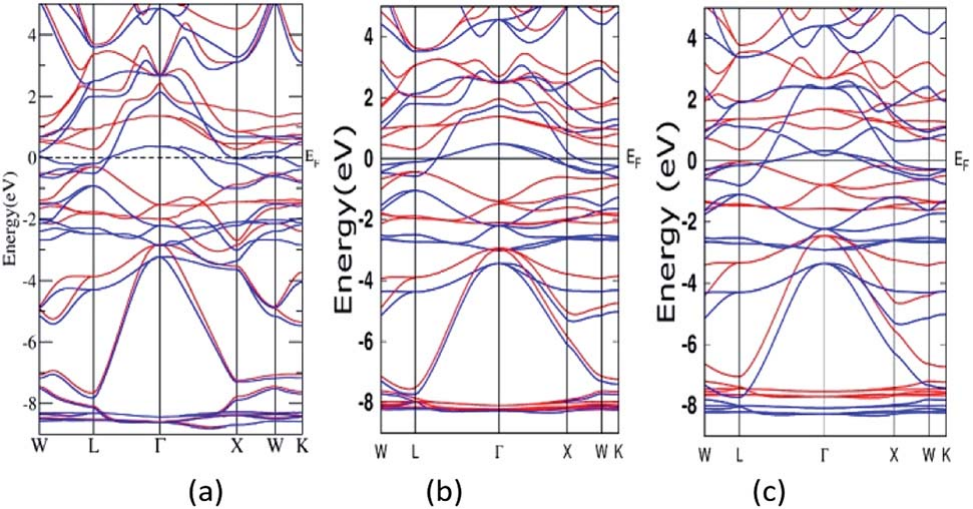
Band structure for Mn_2_ZnSi as calculated from (a) FP-LAPW and (b) PW-PP and for (c) Mn_2_ZnGe. (Colour scheme: blue colour for majority states and red colour for minority states).

A semi relativistic calculation of the energy bands of Mn_2_ZnSi along the high symmetric directions of the Brillouin zone (BZ) was performed to understand the ground state electronic properties using the FP-LAPW and PW methods (Fig. 2a and b). The energy bands obtained from the two methods had a similar profile and were consistent with the previous reports.^29,30^ Further, from Fig. 2, one can note that the majority spin channel had dense bands crossing *E*_F_, which was set to 0.0 eV, giving a metallic nature for majority spin, whereas minority spin had a discontinuity with a distinct energy gap (*E*_g_) at *E*_F_, showing a semiconducting behaviour. The valence band maximum and conduction band minimum was found to be at the L symmetry point of the BZ, producing a direct band *E*_g_ at this point. The *E*_g_ so obtained by the PW and FP-LAPW methods were 0.61 eV and 0.57 eV, respectively, with the values being in qualitatively good agreement. Kervan *et al.* have reported a direct *E*_g_ of 0.48 eV at the L symmetry point of the BZ in minority spin,^29^ which is consistent with the present report. In addition, the present FP-LAPW-estimated *E*_g_ at *a* = 5.79 Å is in close agreement with the previous reports.^32^ The energy bands and *E*_g_ values so obtained are also comparable to the values reported by Bhat *et al.*^30^ In the case of Mn_2_ZnGe, the obtained *E*_g_ in the present study at *a* = 5.93 Å is 0.39 eV, which is inconsistent with that of 0.21 eV reported by Wei *et al.* at an optimized lattice constant of 5.75 Å.

**Fig. 3:**
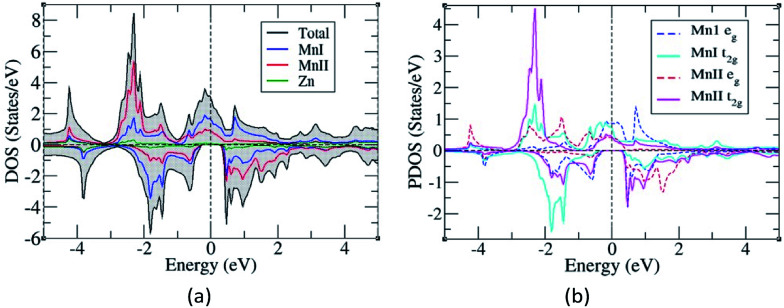
(a) Density of states and (b) Partial density of states of Mn_2_ZnSi(Mn_2_ZnGe).

The total and partial density of states were also studied to understand their energy bands (Fig. 3), and since the electronic structures of Mn_2_ZnSi and Mn_2_ZnGe have a similar profile, we plotted only for one material. From the analysis of the density of states (DOS) for Mn_2_ZnSi(Ge), it is clear that the asymmetry in the DOS of majority and minority spins was due to the asymmetry in the partial density of states (PDOS) of Mn atoms (Fig. 3b). For majority spin, the DOS across the *E*_F_ was arising because of d_eg_ and d_t2g_ states of the MnI atom, whereas −3 eV below *E*_F_ was arising from d_t2g_ states of the MnII atom. Likewise, in the minority spin, the DOS above *E*_F_ was because of the d electrons of MnII atoms, whereas below *E*_F_, it was arising from d_t2g_ states of MnI atoms. There exists a weaker hybridization between Mn and Zn atoms as their peaks are at different positions. Moreover, similar to *E*_F_, the DOS is mainly because of the Mn atoms, which are responsible for the formation of *E*_g_ and dominates the overall electronic properties of the material. The contribution of Si or Ge around *E*_F_ is insignificant and mainly in the region of −5 eV to −3 eV, which is not visible in the DOS plot (Fig. 3).

The occurrence of a bandgap in the cubic inverse full Heusler compounds has been discussed by Skaftouros *et al.*^11^ The Zn atom has completely filled d orbitals that contribute to the core region (−9 eV to −8 eV). The p states of Si(Ge) contribute to the lower valence region, *i.e.*, from −5 eV to −3 eV. The region close to *E*_F_ arises from the Mn atoms due to the interaction of MnI and MnII atoms. Mn atoms have tetrahedral symmetry; therefore, the 3 d_t2g_ orbitals of MnI hybridize with the 3 d_t2g_ orbitals of MnII atoms, giving 3 d_t2g_ bonding and 3 d_t2g_ anti-bonding orbitals. In the same fashion, the 2 d_eg_ orbitals of MnI atoms hybridize with the 2 d_eg_ orbitals of the MnII atoms, again giving 2 d_eg_ bonding and 2 d_eg_ anti-bonding orbitals. Thus, we have five bonding and five anti-bonding d orbitals with *E*_F_ just falling in between the two bands formed by these orbitals.

**Fig. 4:**
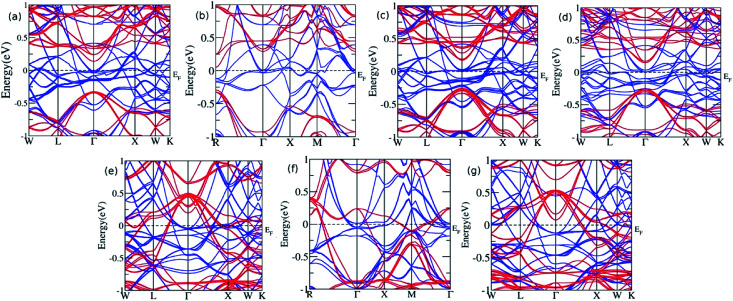
The band structure for Ge doping at varying concentration (a) *x* = 0.125 (b) *x* = 0.25 (c) *x* = 0.375 (d) *x* = 0.5 (e) *x* = 0.625 (f) *x* = 0.75, and (g) *x* = 0.875.

Furthermore, the sample material was doped with a slightly bigger Ge atom in the varying concentration. The presence of Ge in the 0.125, 0.25, 0.375, and 0.5 leads to similar results with a continuous band across *E*_F_, thus showing metallic nature for majority spin, while a direct bandgap of 0.47 eV, 0.56 eV, 0.43 eV, and 0.40 eV was observed for *x* = 0.125, 0.25, 0.375, and 0.5, respectively, in the minority spin. At the Ge concentration of 0.625, 0.75, and 0.875, the band profile reflects their metallic nature (Fig. 4). Moreover, the *E*_g_ value decreases linearly with the increase in the Ge concentration, and shows the metallic character for highest concentration, which is consistent with the previous report published by Hamad *et al.*^36^ describing Fe_2_MnSi_(1−*x*)_Ge_*x*_, where the doping at *x* = 0.75 Ge results in the loss of HM, which is otherwise present for *x* = 0.25 and 0.50. The partial substitution of Ge in Si creates a discrete energy level below the conduction band edge and broadens the energy levels and hence, the *E*_F_ shifts towards the conduction band edge, which results in the *n*-type semiconducting nature of the alloys. However, for the sufficiently high doping of Ge, the conduction and valence band overlap with each other, and *E*_g_ vanishes.

**Fig. 5:**
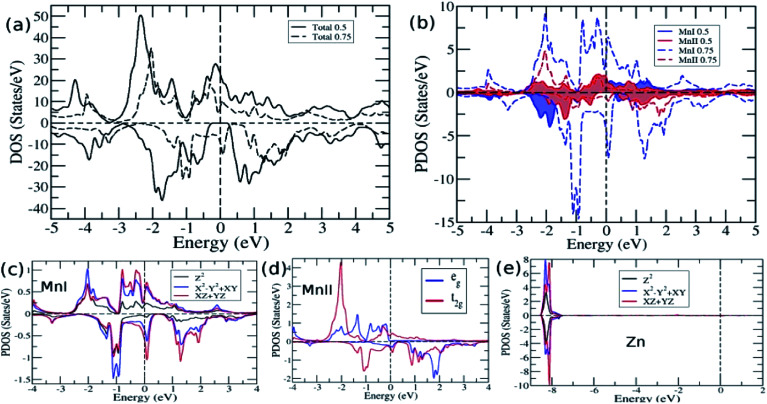
(a) Total DOS for *x* = 0.5 and 0.75 (b) DOS of MnI and MnII for *x* = 0.5 and 0.75 and PDOS for *x* = 0.75 of (c) MnI (d) MnII and (e) Zn.

To further analyse the electronic structure and to understand the cause for the loss of HM for higher Ge concentration, we plotted the total DOS in Fig. 5a for *x* = 0.5 and 0.75. The DOS reflects the features observed in bands, namely, HM for 0.5 Ge and metallic for 0.75 Ge. The two DOS almost appear the same in nature with a shift along the energy axis, except for the appearance of a flat valley between two peaks at −1 eV and 0 eV for *x* = 0.75, which is absent between the two peaks at −1.75 and −0.65 for *x* = 0.5. The flat valley for *x* = 0.75 appears to originate from the slightly lower adjacent peak at −1 eV for *x* = 0.5, which is absent for *x* = 0.75, thus pushing the peak towards EF and eventually crossing *E*_F_. This can be understood from the comparison of DOS of MnI and MnII for *x* = 0.5 and 0.75 (Fig. 5b). The solid lines for MnI and MnII for *x* = 0.5 show the asymmetry in their DOS and peaks at different positions. However, in the case of *x* = 0.75, the MnI and MnII peaks appear almost in the same place but with different strengths.

As explained earlier, the *E*_g_ in the minority spin was due to the hybridization between the d bands of MnI and MnII with tetrahedral symmetry (*T*_d_) that leads to the splitting of d bands into doubly degenerate e_g_ and triply degenerate t_2g_ bands (Fig. 3b). However, on higher doping of Ge, the symmetry of d orbitals was further lowered, and both MnI and Zn exhibited the *D*_3h_ symmetry (Fig. 5c and e), while MnII maintained the *T*_d_ symmetry (Fig. 5d). The *D*_3h_ symmetry splits the d orbitals into three bands, namely, singlet Z^2^ and two doubly degenerate X^2^-Y^2^, XY, and XZ, YZ bands. Moreover, due to the difference in the symmetry of MnI and MnII atoms, there is a weaker hybridization between these atoms, resulting in the loss of *E*_g_ in minority spin.

**Fig. 6:**
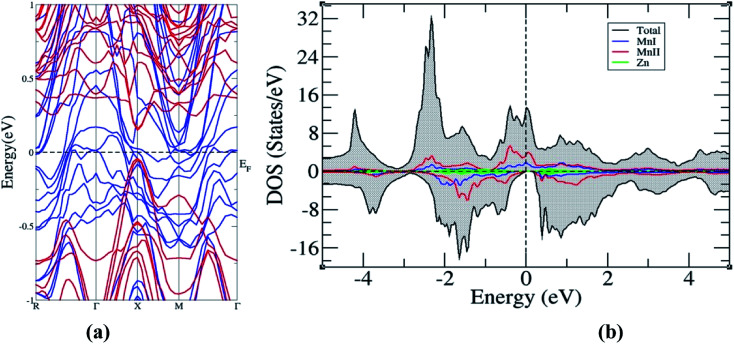
(a) Band structure and (b) density of states of superlattice Mn_2_ZnSi/Mn_2_ZnGe.

The electronic structure of the superlattice, presented in Fig. 6, shows the preservation of the HM character of the bulk Mn_2_ZnSi(Ge), and on comparative analysis with the band structures of Mn_2_ZnSi and Mn_2_ZnGe, we found that the bandgap reduces to 0.22 eV with the shift in the bandgap from L to X. The reduction in the bandgap can be understood by comparing the minority band edge below and above *E*_F_ of the bulk (Fig. 3a and b) and superlattice (Fig. 6b), as suggested by Ghaderi *et al.*^60^ There is a contribution from both MnI and MnII in the minority valence band edge below *E*_F_ in the case of bulk Mn_2_ZnSi(Ge). However, in the case of a superlattice of Mn_2_ZnSi/Mn_2_ZnGe, the contribution comes only from MnII atoms. This is due to the lowering of the coordination number of atoms at the interface of a superlattice with respect to the atoms of the bulk, which enhances the exchange and thus increases the splitting of majority and minority DOS, as observed by the number of finer peaks in Fig. 6b.

The calculated individual and total magnetic moments for pure and Ge-doped compounds are presented in Table 3, which depicts that the magnetic behaviour mainly arises from the Mn atoms as the magnetic moment of Zn and Si (Ge) are comparatively minuscule. The total magnetic moments of Mn_2_ZnSi(Ge) per unit formula is 2.00 *μ*_B_, in accordance with the Slater–Pauling (SP) rule^11^ of *M*_T_ = *Z*_T_ – 28, where *M*_T_ is the total magnetic moment and *Z*_T_ is the number of valence electron of the material. This rule is in accordance with the hybridization mechanism explained above for the formation of *E*_g_. The individual magnetic moments of MnI and MnII are aligned antiparallel to each other, which can be explained from their asymmetric spin-polarized DOS and PDOS of Mn_2_ZnSi (Fig. 3a and b).^29,30^ As a result of unoccupied 3d spin-up states of MnI and 3d spin-down states of MnII, the magnetic moments of the two Mn atoms are aligned anti-parallel to each other.

**Table 3:** Calculated total and individual magnetic moments of pure and doped compounds

Magnetic moment in (*μ*_B_)		MnI	MnII	Zn	Si	Ge	Total
Mn_2_ZnSi	Present work	−1.279	3.213	0.009	0.059	—	2.00
	Others	−0.778 (ref. 29)	2.664	0.268	0.032	—	2.00
		−0.741 (ref. 30)	2.596	0.020	0.032	—	2.00
Ge concentration (*x*)	0.125	−0.823	2.703	0.028	0.035	0.032	15.969
	0.25	−0.654	2.573	0.026	0.026	0.031	7.993
	0.375	−0.064	2.583	0.025	0.025	0.03	16.177
	0.5	−0.542	2.49	0.023	0.026	0.03	16.054
	0.625	1.248	2.185	−0.009	−0.001	−0.03	27.8
	0.75	0.73	2.543	−0.012	−0.099	0.007	13.282
	0.875	1.012	2.753	−0.015	−0.005	0.003	30.079
Mn_2_ZnGe	Present work	−1.181	2.629	−0.021	—	0.059	1.98
	Others	0.43 (ref. 31)	−2.4	−0.02	—	−0.01	−2.00

**Fig. 7:**
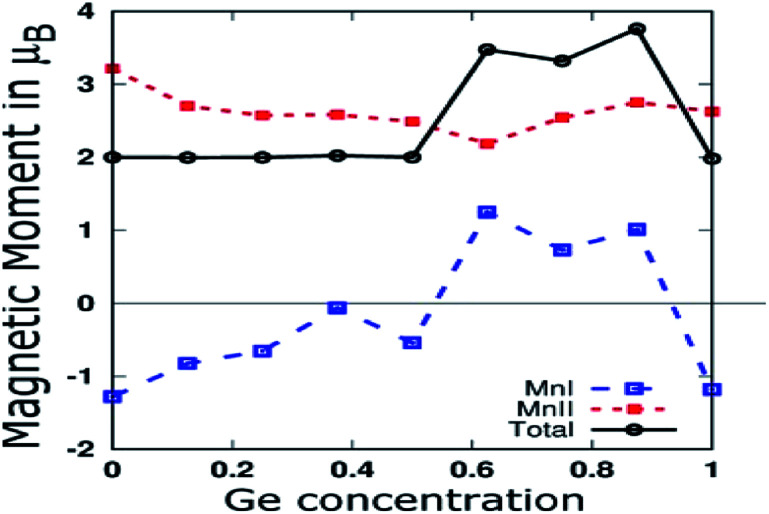
MnI and MnII Magnetic moments, and total magnetic moment per formula unit of partial Ge doped compounds.

As shown in Fig. 7, we plotted the variation of individual magnetic moments of MnI, MnII, and total magnetic moment per formula unit of partially Ge doped compounds as a function of Ge concentration. The total magnetic moment per formula unit remains almost 2.0 *μ*_B_ up to 0.5 Ge, in accordance with the SP rule of *M*_T_ = *Z*_T_ − 28, confirming the HM for these dopings. The magnetic moment of MnII remains positive throughout the concentration of Ge, whereas it remains negative up to 0.5 Ge and thereafter, it becomes positive in the case of MnI. This can be explained in terms of Mn–Mn interaction, which depends upon the Mn–Mn distance. Mn magnetic moments couple anti-parallel to each other for small Mn–Mn distances and couple parallel to each other for large Mn–Mn distances, which is consistent with the report by Bethe and Slater.^61^ Hence, increasing the Ge concentration expands the crystal size, which in turn increases the Mn–Mn distances and favours the parallel alignment of the Mn moment, as reported by Galanakis *et al.* for Ni_2_MnAl.^62^

## 4. Conclusions

We have investigated the ground state electronic and magnetic properties of Mn_2_ZnSi_(1−*x*)_Ge_*x*_ (*x* = 0, 0.125, 0.25, 0.375, 0.5, 0.625, 0.75, 0.875, and 1.0) Heusler alloys and Mn_2_ZnSi/Mn_2_ZnGe superlattice using DFT-based PW-PP and FP-LAPW methods. The stable ground state structures for these alloys were found to energetically favour the magnetic phase over the non-magnetic phases. The results so obtained using PW-PP and FP-LAPW were in good agreement with the previous studies. The elastic constants predicted the mechanical stability of these alloys at ambient conditions. The spin-polarized electronic band structures and DOS were also studied for pure, doped, and superlattice compounds. The analysis of the electronic band structures predicted half-metallicity with 100% spin-polarized electrons at *E*_F_ for the pure Heusler alloys. However, the partial doping of Ge resulted in HM for *x* = 0.125 to 0.5 and a higher concentration leads to a metallic state. A deeper analysis of DOS and PDOS for HM and metallic phases revealed the change in the symmetry of MnI and Zn from *T*_d_ to *D*_3h_ as the cause of the transition from HM to metallic nature. The PDOS also revealed the antiparallel coupling of Mn atoms and the interaction of the neighbouring Mn atoms, which is the cause of *E*_g_. The electronic structure for the superlattice of Mn_2_ZnSi/Mn_2_ZnGe also conserves the HM of bulk Mn_2_ZnSi(Ge). The total magnetic moments per formula unit for these alloys were close to the integral value and were in good agreement with the Slater–Pauling rule of *M*_T_ = *Z*_T_ − 28. Thus, the presence of a half-metallic bandgap with 100% spin-polarized electrons at *E*_F_ and the ferrimagnetic ordering of Mn atoms resulting in low saturation magnetic moments make these Mn_2_ZnSi_(1−*x*)_Ge_*x*_ Heusler alloys suitable materials in spintronic applications.

## Conflicts of interest

There are no conflicts to declare.

## Supplementary Material
